# Epidemiology of *Klebsiella pneumoniae* bloodstream infections in a teaching hospital: factors related to the carbapenem resistance and patient mortality

**DOI:** 10.1186/s13756-016-0145-0

**Published:** 2016-11-17

**Authors:** Lijun Tian, Ruoming Tan, Yang Chen, Jingyong Sun, Jialin Liu, Hongping Qu, Xiaoli Wang

**Affiliations:** 1 Department of Critical Care Medicine, Ruijin Hospital, Shanghai Jiao Tong University School of Medicine, No.197 Ruijin ER Road, Shanghai, 200025 China; 2 Department of Clinical Microbiology, Ruijin Hospital, Shanghai Jiao Tong University School of Medicine, No.197 Ruijin ER Road, Shanghai, 200025 China

**Keywords:** *Klebsiella pneumoniae*, Bloodstream infection, Epidemiology, Carbapenem resistance, Mortality

## Abstract

**Background:**

Although *Klebsiella pneumoniae* bloodstream infections (KP-BSIs) have recently attracted attention due to an alarming raise in morbidity and mortality, there have been few reports on the epidemiology of KP-BSIs in mainland China. We sought to describe the epidemiological, microbiological, and clinical characteristics of KP-BSIs, focusing on the risk factors of carbapenem resistance and patient mortality.

**Methods:**

A retrospective analysis of WHONET data of KP-BSI patients admitted to a teaching hospital in Shanghai, China, between January 1, 2011 and December 31, 2015 was performed, and the annual percentage of patients with carbapenem-resistant *K. pneumoniae* (CRKP) was determined. Risk factors related to the carbapenem resistance and patient mortality were analyzed using binary logistic regression model. The genetic relatedness of CRKP strains isolated from intensive care unit (ICU) patients was determined by pulsed-field gel electrophoresis (PFGE).

**Results:**

A total of 293 incidences of KP-BSIs were identified in a 5-year period, 22.18% of these (65/293) were CRKP strains, and the proportion of CRKP-BSI in ICU was 59.62% (31/52), equaling the levels observed in the epidemic regions. A number of KP-BSIs (114), obtained from January 1, 2014, to December 31, 2015, were further investigated. Skin and soft tissue infection source (odds ratio [OR] 26.63, 95% confidence interval [CI] 4.8–146.8) and ICU-acquired infection (OR 5.82, 95% CI 2.0–17.2) was shown to be powerful risk factors leading to the development of CRKP-BSI. The crude 28-day mortality rates of KP-BSI and CRKP-BSI patients were 22.8% and 33.3%, respectively. Lung as the probable source of infection (OR 4.23, 95% CI 1.0–17.3), and high Sequential Organ Failure Assessment (SOFA) score (OR 1.40, 95% CI 1.2–1.6) were strong prognostic factors determining crude 28-day KP-BSI mortality rates. PFGE analysis demonstrated that 10/11 random CRKP isolates in ICU belonged to the same clonal type.

**Conclusions:**

During the study period, we observed a significant increase in the occurrence of CRKP infections among the identified KP-BSIs in our hospital and especially in ICU, and we demonstrated that carbapenem resistance is associated with the increased mortality of KP-BSI patients.

## Background

*Klebsiella pneumoniae* (KP) is an important pathogen responsible for severe diseases such as septicemia, pneumonia, urinary tract infections, and soft tissue infections, and this pathogen is mainly associated with community- and hospital-acquired infections [1]. Nielsen *et al*. reported that *K. pneumoniae* is the second most common cause of gram-negative bloodstream infections (BSIs), after *Escherichia coli,* in adult population [2]. However, the incidence of KP-BSIs had exceeded the incidence of *E. coli*-caused BSIs in recent studies investigating intensive care unit (ICU) patients [3, 4]. High mortality rates, ranging from 20 to 40%, were reported for patients with KP-BSI [5], but this incidence was reported to rise up to 67.6% for ICU patients [3].

Bacterial and host factors, as well as different treatment regimens, most likely influence the outcomes of patients with KP-BSI. Carbapenem-resistant *K. pneumoniae* (CRKP) infections may be associated with an increased rate of treatment failure and death [6], and the dramatic increase in the occurrence of CRKP worldwide aggravates this situation further [7]. Furthermore, older age, nosocomial infections, intensive care interventions, disease severity, and several comorbidities were reported to represent host factor contributing to the increased mortality rates of KP-BSI patients [3, 5, 8, 9]. Additionally, β-lactams were the first-line antibiotics selected for the treatment of KP-BSI patients, however, with the increase in the number of *K. pneumoniae* isolates producing extended-spectrum β-lactamase, the use of carbapenems increased as well. More importantly, CRKP isolates were shown to be resistant to a variety of antibiotics, and to have the ability to hydrolyze carbapenems, leaving few effective treatment options. The use of combination therapy and appropriate empirical treatment may provide useful prognostic information in these patients [10–12].

Although the high mortality rates have been extensively reported worldwide, to date, few studies have investigated the epidemiology of KP-BSIs in mainland China. The aim of this study was to describe the epidemiological, microbiological, and clinical characteristics of KP-BSIs in patients admitted to a teaching hospital in Shanghai, China, focusing on the risk factors related to carbapenem resistance and patient mortality.

## Methods

### Study design and population

Study design and all experimental procedures were approved by the Institutional Review Board of Ruijin Hospital, an 1800-bed tertiary care university teaching hospital in Shanghai, China. WHONET data provided by clinical microbiology laboratory were used to identify all KP-BSI patients between January 1, 2011, and December 31, 2015. Only the first KP-BSI reported for each patient was included in our analyses. These data were used to evaluate the rate of CRKP-BSIs.

We chose 114 incidences of KP-BSIs that occurred between January 1, 2014, and December 31, 2015 for further investigation. Clinical and microbiological characteristics and treatment outcomes were retrieved from the medical records by two experienced ICU medical doctors. The information about the patient demographics (age, gender), comorbidities (diabetes mellitus, chronic renal failure, chronic liver disease, biliary tract disease, congestive heart failure, chronic obstructive pulmonary disease, malignancy, and immunosuppression), probable source of infection, prior healthcare exposure conditions, such as major surgery in the past 30 days and the receipt of any antibiotic during >48 h in the past 30 days, was collected. Additionally, the general state of patients on the onset of BSI was assessed, such as acute kidney injury, septic shock, use of mechanical ventilation, use of renal replacement therapy (RRT), and the removal of the probable infectious source. Comorbid conditions were also determined using the Charlson comorbidity index (CCI) as previously described [13], and the severity of illness during the onset of BSI was calculated by the Pitt bacteraemia score (PBS) and Sequential Organ Failure Assessment score (SOFA score) [14, 15]. Furthermore, the appropriate empirical antimicrobial therapies described by Zarkotou were considered as well [10]. We investigated attributable and crude in-hospital mortality rates and 28-day mortality rates of patients, in order to assess the treatment outcomes. Total length of hospital stay (LOS) and LOS after BSI were evaluated, while the primary outcome in this study was the crude 28-day mortality rate.

### Definitions

BSI was defined according to the Centers for Disease Control and Prevention guidelines (available at: http://www.cdc.gov/nhsn/pdfs/pscmanual/17pscnosinfdef_current.pdf). BSI onset was defined as the collection date of isolate. Nosocomial infection was defined as an infection that occurred more than 48 h after the admission of the patient to the hospital or an infection that existed in patients who had been admitted to other hospitals in 2 weeks before the current admission [16]. ICU-acquired BSI was defined as the first positive blood culture identified more than 2 days after ICU admission (without a prior positive blood culture with the same pathogen for at least 30 days). The probable infectious source was determined on the basis of the microbiological results and the analysis by 2 physicians. When no source was identified, primary BSI was recorded. Appropriate empirical antimicrobial treatment refers to the administration of *in vitro* active antimicrobials against the study isolates, within ≤24 h from the BSI onset [10]. Septic shock was defined as sepsis associated with organ dysfunction and persistent hypotension despite volume replacement, and acute kidney injury (AKI) was defined using the established criteria [17, 18]. Crude mortality was defined as death occurring after the collection of the first blood culture positive for *K. pneumoniae*. Mortality attributable to BSI was defined by clinical evidence of active infection and positive cultures, or when death occurred as the result of organ failure that developed or deteriorated during the onset of infection [19].

### Microbiology

Vitek 2 automated system (bioMérieux, Marcy l’Etoile, France) was used for isolate identification and antimicrobial susceptibility testing. Minimum inhibitory concentrations (MICs) were classified according to breakpoints established by the Clinical and Laboratory Standards Institute (CLSI2014) [20]. *E. coli* ATCC 25922 was used as a quality control reference strain. The genetic relatedness of CRKP strains obtained from ICU patients was determined by pulsed-field gel electrophoresis (PFGE) [21]. PFGE patterns were interpreted using the criteria proposed by Tenover *et al*. [22].

### Statistical analysis

All results are expressed as the mean ± standard deviation (SD) or median (interquartile range [IQR]) (continuous variables) or as percentages of the group from which they were derived (categorical variables). Student’s *t*-test and Mann-Whitney *U*-test were used to compare normally and non-normally distributed continuous variables, respectively. Categorical variables were compared using the χ^2^ test or Fisher’s test, as appropriate. Odds ratios (ORs) and 95% confidence intervals (CIs) were calculated for all emerging associations. Multivariate analysis was used to identify independent predictors by applying binary logistic regression. Variables with *p*-value <0.05 in univariate testing were incorporated into the model using a forward stepwise approach, and *p*-value <0.05 was set as the limit for the retention of the variables in the model. The goodness of fit for our logistic regression model was assessed with Hosmer-Lemeshow test. In multivariate analysis, comorbidities were substituted using the CCI, and hospital events on the onset of BSI were replaced by PBS and SOFA score. Differences were considered to be significant for *p* < 0.05. Data were analyzed with the IBM SPSS Statistics for Windows (version 19.0). All susceptibility data were analyzed using WHONET, version 5.6.

## Results

### Increased rates of CRKP-BSIs, especially among ICU patients

**Fig. 1:**
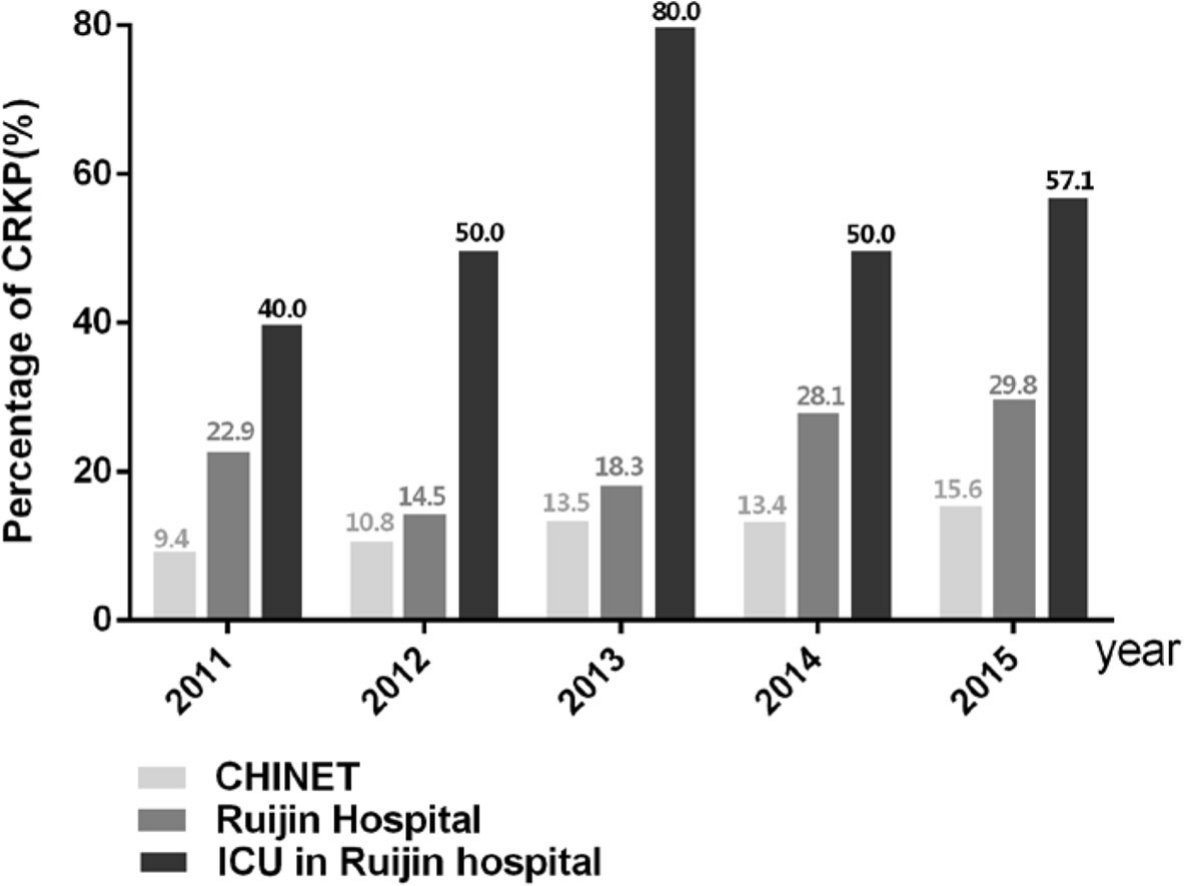
CRKP among identified KP-BSIs from January 2011 to December 2015. Light grey bar, percentage of CRKP from CHINET; dark grey bar, percentage of CRKP-BSI in Ruijin Hospital from WHONET; black bar, percentage of CRKP-BSI in ICU of Ruijin Hospital from WHONET

KP-BSIs were collected and analyzed using WHONET from January 1, 2011, to December 31, 2015 in our hospital. During this period, 293 episodes of KP-BSI were identified, and 22.18% (65/293) of these cases were CRKP isolates, while 47.7% (31/65) of the CRKP isolates were obtained from ICU. The annual rate of CRKP in clinical isolates in China, obtained from the CHINET surveillance of bacterial resistance [23], and the percent of CRKP-BSI determined in this study are presented in Fig. 1. The percentage of CRKP-BSIs in our hospital significantly increased from 14.5% in 2012 to 29.8% in 2015, together with the percentage growth from 40% in 2011 to 57.1% in 2015 in the isolates from ICU patients, with the highest rate obtained in 2013, up to 80%. A similar trend, with the rate of CRKP rising from 9.4% in 2011 to 15.6% in 2015 was observed in data obtained from the CHINET surveillance program.

We demonstrated that the occurrence of CRKP strains causing KP-BSIs since 2011 is increasing, and the annual rates of these infections in the hospital included in this study are much higher than the national average. Additionally, the proportion of CRKP-BSIs in ICU patients was 59.62% (31/52), which is significantly higher than 14.11% (34/241) of the patients with these infections in non-ICU during this 5-year period (*p* < 0.001).

### Risk factors associated with the development of CRKP-BSIs

Because of the significant increase in the number of CRKP-BSI cases in our hospital since 2014 and the increased accuracy of the medical records, 114 incidences of KP-BSIs that occurred between January 1, 2014, and December 31, 2015, were further investigated. The mean age of these 114 patients was 56 years (median: 67, range: 12–90 years), and 64.9% (74/114) of the patients were male. Most of the KP-BSIs (86.0%) were nosocomial infections, and among them, 26 cases (22.8%) were acquired at ICU. Most common infection sources were primary bloodstream infections (23.7%), and intra-abdominal (42.1%) and lung (14.0%) infections. The median CCI was 3 (IQR, 2-6) with the most frequent comorbidities being malignancy (51.8%), biliary tract disease (30.7%), and diabetes mellitus (26.3%). The median PBS and SOFA score were 1 (IQR, 1–2.25) and 3 (IQR, 1–6), respectively.

To identify the clinical characteristics affecting the development of CRKP-BSIs, we compared the clinical characteristics of patients with CRKP-BSIs and CSKP-BSIs (Table 1). The factors determined to be significantly associated with CRKP-BSIs, using the univariate analysis, included nosocomial infection, ICU-acquired infection, probable infection source from skin and soft tissue, prior exposure to antimicrobial therapy in the past 30 days, and high PBS. In contrast, diabetes mellitus, malignancy, and high CCI were common in patients with CSKP-BSIs. Using multivariate analysis, skin and soft tissue infection source (OR 26.63, 95%CI 4.8–146.8) and ICU-acquired infection (OR 5.82, 95%CI 2.0–17.2) was determined to be independent factors for CRKP-BSI development.

**Table 1:** Comparison of clinical characteristics between CRKP-BSI and CSKP-BSI patients

Variable	Total (*n* = 114) n(%)	CRKP (*n* = 33) n(%)	CSKP (*n* = 81) n(%)	Univariate analysis		Multivariate analysis	
				OR(95% CI)	*p*-value	OR(95% CI)	*p*-value
General variables							
Male sex	74(64.9)	22(66.7)	52(64.2)	1.12(0.5-2.6)	0.802		
Age,years, mean ± SD	56.37 ± 16.36	51.70 ± 16.26	58.27 ± 16.11	NA	0.051		
Poly-microbial BSI	11(9.6)	6(18.2)	5(6.2)	3.38 (1.0–12.0)	0.076		
Nosocomial infection	98(86.0)	33(100)	65(80.2)	NA	0.005	-	-
ICU acquired infection	26(22.8)	14(42.4)	12(14.8)	4.24(1.7–10.7)	0.001	5.82(2.0–17.2)	0.002
Comorbidities							
Diabetes mellitus	30(26.3)	3(9.1)	27(33.3)	0.20(0.1–0.7)	0.008		
Chronic renal failure	8(7.0)	3(9.1)	5(6.2)	1.52(0.3–6.8)	0.689		
Chronic liver disease	20(17.5)	5(15.2)	15(18.5)	0.79(0.3–2.4)	0.668		
Liver cirrhosis	11(9.6)	2(6.1)	9(11.1)	0.52(0.1–2.5)	0.506		
Biliary tract disease	35(30.7)	9(27.3)	26(32.1)	0.79(0.3–1.9)	0.612		
Congestive heart failure	9(7.9)	4(12.1)	5(6.2)	2.10(0.5–8.4)	0.280		
Chronic obstructive pulmonary disease	3(2.6)	1(3.0)	2(2.5)	1.23(0.1–14.1)	1		
Malignancy	59(51.8)	12(36.4)	47(58.0)	0.41(0.2–1.0)	0.036		
Immunosuppression	26(22.8)	10(30.3)	16(19.8)	1.77(0.7–4.4)	0.223		
CCI,median (IQR)	3(2–6)	3(0–4)	4(2–6)	NA	0.005	-	-
Probable source of infection							
Lung	16(14.0)	5(15.2)	11(13.6)	1.14(0.4–3.6)	0.776		
Urinary	1(0.9)	0(0)	1(0.9)	NA	1		
Intra-abdominal	48(42.1)	12(36.4)	36(44.4)	0.71(0.3–1.6)	0.428		
Liver abscess	6(5.3)	1(3.0)	5(6.2)	0.48(0.1–4.2)	0.671		
Skin and soft tissue	12(10.5)	10(30.3)	2(2.5)	17.17(3.5–84.0)	<0.001	26.63(4.8–146.8)	<0.001
Catheter	10(8.8)	5(15.2)	5(6.2)	2.71(0.7–10.1)	0.150		
Primary	27(23.7)	5(15.2)	22(27.2)	0.48(0.2–1.4)	0.171		
Hospital events prior to onset of BSI							
Exposure to antimicrobial therapy ^a^	74(64.9)	30(90.9)	44(54.3)	8.41(2.4–29.8)	<0.001	4.04(1.0–16.5)	0.052
Surgery ^a^	62(54.4)	21(63.6)	41(50.6)	1.71(0.7–3.9)	0.206		
Events on the onset of BSI							
SOFA score,median (IQR)	3(1–6)	4(1–7)	3(1–5)	NA	0.098		
PBS,median (IQR)	1(1-2.25)	2(1–5)	1(0–2)	NA	<0.001	-	-

^a^in the past 30 days prior to onset of BSI

*SD*, standard deviation, *IQR* interquartile range, *OR* odds ratio, *CI* confidence interval, *ICU* intensive care unit, *BSI* bloodstream infection, *CRKP* carbapenem-resistant *Klebsiella pneumoniae*, *CSKP* carbapenem-sensitive *Klebsiella pneumoniae*, *LOS* length of stay, *CCI* Charlson comorbidity index, *PBS* Pitt bacteraemia score, *SOFA score* sequential organ failure assessment score, *NA* non-applicable

### Comparison of antimicrobial therapies and treatment outcomes between CRKP-BSI and CSKP-BSI patients

Antimicrobial therapies and outcomes of patients with KP-BSIs are presented in Table 2. Patients with CSKP-BSI were more likely to receive appropriate empirical therapy than the patients with CRKP-BSI. Patients with CRKP-BSI had longer total LOS compared with the patients with CSKP-BSI (median [IQR], 50 [28-83] *vs*. 24 [16.5-51], *p* < 0.001). The crude 28-day mortality and in-hospital mortality of KP-BSI patients were 22.8% (26/114) and 26.3% (30/114), respectively. Crude in-hospital mortality was higher in patients with CRKP-BSIs than in those with CSKP-BSIs (42.4% *vs*. 19.8%, respectively, *p* = 0.013), and this difference existed when crude 28-day mortality was compared (33.3% *vs.* 18.5%, respectively, ?>*p* = 0.087). Additionally, attributable 28-day mortality and in-hospital mortality of KP-BSI patients was 21.1% (24/114) and 24.6% (28/114), respectively. Similarly, in CRKP-BSI patients, the attributable 28-day mortality and in-hospital mortality were significantly higher than in CSKP-BSI patients (33.3% *vs*. 16%, *p* = 0.04; 42.4% *vs*. 24.6%, *p* = 0.005, respectively).

**Table 2:** Comparison of antimicrobial therapies and treatment outcomes between CRKP-BSI and CSKP-BSI patients

Treatment and outcomes	Total (*n* = 114)	CRKP (*n* = 33)	CSKP (*n* = 81)	*p*-value
Appropriate empirical antimicrobial therapy	78(68.4)	9(27.3)	69(85.2)	<0.001
LOS after BSI onset,Days, median (IQR)	18.5(9–31)	24(9.5–51)	15(8.5–28)	0.066
Total LOS,days, median (IQR)	31.5(19.75–62.75)	50(28–83)	24(16.5–51)	0.001
Mortality rate				
Crude 28-day mortality n(%)	26(22.8)	11(33.3)	15(18.5)	0.087
Crude in-hospital mortality n(%)	30(26.3)	14(42.4)	16(19.8)	0.013
Attributable 28-day mortality n(%)	24(21.1)	11(33.3)	13(16.0)	0.04
Attributable in-hospital mortality n(%)	28(24.6)	14(42.4)	14(24.6)	0.005

*IQR* interquartile range, *BSI* bloodstream infection, *CRKP* carbapenem-resistant Klebsiella pneumoniae, *CSKP* carbapenem-sensitive Klebsiella pneumoniae, *LOS* length of stay

### Identification of clinical and microbiological characteristics associated with crude 28-day mortality

A comparison of clinical and microbiological characteristics of the patients enrolled in this study is presented in Table 3. We aimed to identify the risk factors associated with crude 28-day mortality of KP-BSI patients. In the univariate analysis, ICU-acquired KP-BSI was determined to be a risk factor (OR 2.81, 95% CI 1.1–7.3) and, when all comorbidities were analyzed, chronic liver disease (OR 3.71, 95% CI 1.3–10.3) and congestive heart failure (OR 5.00, 95% CI 1.2–20.2) were shown to be risk factors as well. Probable lung infection (OR 6.13, 95% CI 2.0–18.7) was the source of these bloodstream infections more frequently in non-survivors than in survivors. Moreover, mortality rate was affected by the events on the onset of BSIs, such as acute kidney injury, septic shock, RRT use, and use of mechanical ventilation. Additionally, high SOFA score (median [IQR], 9 [4–13.5] *vs*. 2 [1–4], *p* < 0.001) and high PBS (median [IQR], 5 [2–7] *vs*. 1 [1, 2], *p* < 0.001) were associated the crude 28-day mortality in univariate analyses. There was no statistical difference in hospital events prior to the onset of BSI between survivors and non-survivors. Obtained using multivariate logistic regression analysis, significant risk factors for crude 28-day mortality were lungs as probable source of infection (OR 4.23, 95% CI 1.0–17.3), and high SOFA score (OR 1.40, 95% CI 1.2–1.6), after adjusting for other confounding variables.

**Table 3:** Identification of clinical and microbiological characteristics associated with crude 28-day mortality

Variable	Total (*n* = 114) n(%)	Death (*n* = 26) n(%)	Survivors (*n* = 88) n(%)	Univariate analysis		Multivariate analysis	
				OR(95% CI)	*p*-value	OR(95% CI)	*p*-value
General variables							
Male sex	74(64.9)	20(76.9)	54(61.4)	2.10(0.8–5.8)	0.144		
Age,years, mean ± SD	56.37 ± 16.36	55.54 ± 16.10	56.61 ± 16.52	NA	0.770		
Poly-microbial BSI	11(9.6)	3(11.5)	8(9.1)	1.30(0.3–5.3)	0.711		
Nosocomial infection	98(86.0)	23(88.5)	75(85.2)	1.33(0.3–5.1)	1		
ICU acquired infection	26(22.8)	10(38.5)	16(18.2)	2.81(1.1–7.3)	0.03	-	-
Comorbidities							
Diabetes mellitus	30(26.3)	4(15.4)	26(29.5)	0.43(0.1–1.4)	0.150		
Chronic renal failure	8(7.0)	1(3.8)	7(8.0)	0.463(0.05–3.9)	0.680		
Chronic liver disease	20(17.5)	9(34.6)	11(12.5)	3.71(1.3–10.3)	0.017		
Liver cirrhosis	11(9.6)	5(19.2)	6(6.8)	3.25(0.9–11.7)	0.122		
Biliary tract disease	35(30.7)	8(30.8)	27(30.7)	1.00(0.4–2.6)	1		
Congestive heart failure	9(7.9)	5(19.2)	4(4.5)	5.00(1.2–20.2)	0.028		
Chronic obstructive pulmonary disease	3(2.6)	0(0)	3(3.4)	NA	1		
Malignancy	59(51.8)	11(42.3)	48(54.5)	0.61(0.3–1.5)	0.273		
Immunosuppression	26(22.8)	6(23.1)	20(22.7)	1.02(0.4–2.9)	0.97		
Charlson comorbidity index, median (IQR)	3(2–6)	3(2–6.75)	3(1.25–6)	NA	0.576		
Probable source of infection							
Lung	16(14.0)	9(34.6)	7(8.0)	6.13(2.0–18.7)	0.002	4.23(1.0–17.3)	0.045
Intra-abdominal	48(42.1)	9(34.6)	39(44.3)	0.67(0.3–1.7)	0.379		
Skin and soft tissue	12(10.5)	4(15.4)	8(9.1)	1.82(0.5–6.6)	0.465		
Catheter-related	10(8.8)	2(7.7)	8(9.1)	0.83(0.2–4.2)	1		
Hospital events prior to onset of BSI							
Exposure to antimicrobial therapy ^a^	74(64.9)	20(76.9)	54(61.4)	2.10(0.8–5.8)	0.144		
Surgery ^a^	62(54.4)	15(57.7)	47(53.4)	1.19(0.5–2.9)	0.700		
Events on the onset of BSI							
Acute kidney injury	16(14.0)	12(46.2)	4(4.5)	18.00(5.1–63.8)	<0.001		
Use of renal replacement therapy	8(7.0)	5(19.2)	3(3.4)	6.75(1.5–30.5)	0.015		
Septic shock	28(24.6)	16(61.5)	12(13.6)	10.13(3.7–27.5)	<0.001		
Use of mechanical ventilation	24(21.1)	15(57.7)	9(10.2)	11.97(4.2–33.9)	<0.001		
Removal of the infectious source	33(28.9)	6(23.1)	27(30.7)	0.68(0.2–1.9)	0.453		
SOFA score, median (IQR)	3(1–6)	9(4–13.5)	2(1–4)	NA	<0.001	1.40(1.2–1.6)	<0.001
Pitt bacteraemia score, median (IQR)	1(1–2.25)	5(2–7)	1(1–2)	NA	<0.001	-	-
CRKP	33(28.9)	11(42.3)	22(25.0)	2.20(0.9–5.5)	0.087		
Appropriate empirical therapy	78(68.4)	14(53.8)	64(72.7)	0.44(0.2–1.1)	0.069		

^a^in the past 30 days prior to onset of BSI

*SD* standard deviation, *IQR* interquartile range, *OR* odds ratio, *CI* confidence interval, *ICU* intensive care unit, *BSI* bloodstream infection, *CRKP* carbapenem-resistant *Klebsiella pneumoniae*, *CSKP* carbapenem-sensitive *Klebsiella pneumoniae*, *LOS* length of stay, *CCI* Charlson comorbidity index, *PBS* Pitt bacteraemia score, *SOFA score* sequential organ failure assessment score

### PFGE analysis of random CRKP ICU isolates

**Fig. 2:**
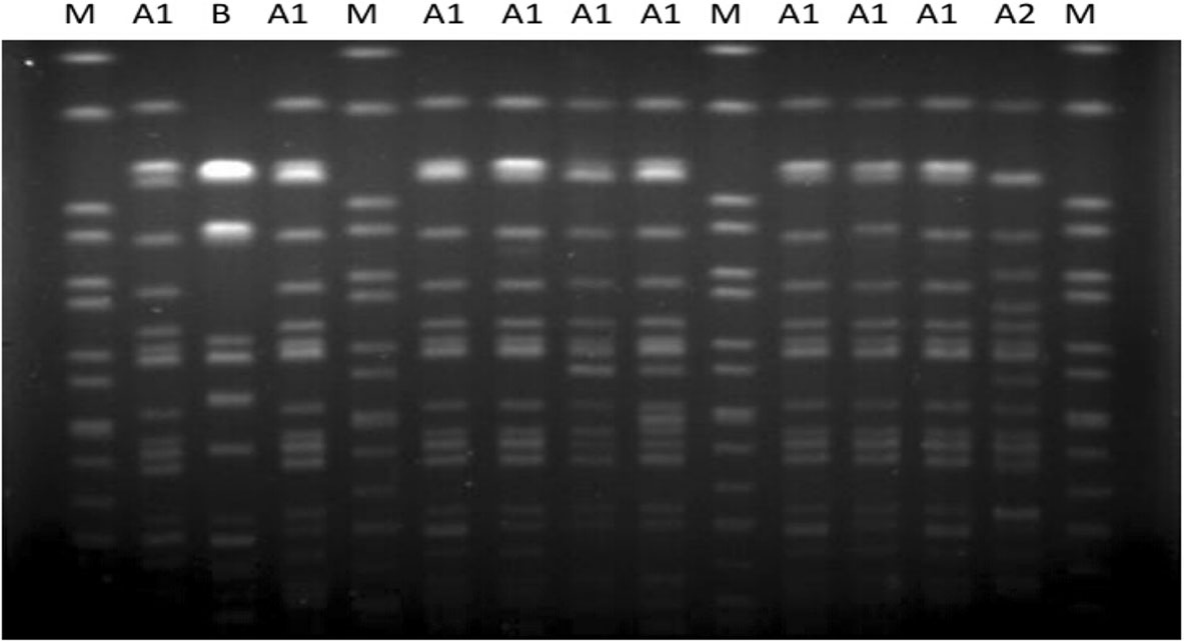
PFGE analysis of random CRKP ICU isolates. Genomic DNA was digested using XbaI enzyme and subjected to PFGE. PFGE patterns were interpreted by using the criteria proposed by Tenover *et al.* [24]. Lane M, a Salmonella serotype Braenderup strain (H9812) was used to normalize migration variation occurring across the gel and to accurately determine sample band sizes

To further determine the genetic relatedness of CRKP strains in ICU, we randomly selected 11 CRKP ICU isolates for PFGE analysis. The obtained results indicated that these isolates belong to two different clonal types (Fig. 2). The predominant clonal type was shown to be A-type (10 isolates [90.9%], including A1-subtype [*n* = 9] and A2-subtype [*n* = 1]).

## Discussion

The increase in the morbidity and mortality of KP-BSIs has attracted a lot of attention recently, together with the worldwide spread of hypervirulent and multidrug-resistant *K. pneumoniae* strains. To the best of our knowledge, this is the largest 5-year epidemiological study of KP-BSIs in mainland China to date. In China, CHINET data showed that the rate of carbapenem resistance of *K. pneumoniae* isolates rose slowly from 3.0% in 2005 to 4.9% in 2009, but a rapid increase in the number of CRKP isolates has been reported since 2010, with the current national average as high as 15.6% [23]. Over the past 10 years, the rate of CRKP has also increased dramatically worldwide [7], and in the areas of high CRKP endemicity, such as the United States, Israel, Italy, and Greece, the percentage of CRKP-BSI was shown to be 18–68% [19, 24–26]. In agreement with these results, we observed an increase in CRKP-BSI rates in our hospital after 2011, and the obtained data shows that these rates are significantly higher than the average rates in China, especially among ICU patients, although the rate of CRKP-BSIs in non-ICU patients increased, rising up to 20% since 2014. Noteworthy, the incidence of CRKP-BSIs in our ICU was higher than 50%, which is similar to the rates observed in the endemic areas [25, 27], however, it may be possible that the analyses focused on the patient vulnerability and disease severity, and several patients were transferred from the endemic CRKP regions of China, such as Zhejiang and Jiangxi provinces [28].

Clinical characteristics associated with CRKP-BSI patients in our study were similar to those reported by other studies, with the exception of probable skin and soft tissue infection source [19, 29, 30]. Jolie *et al*. reported that patients with ≥20% total body surface area burns show a higher risk of bacteremia [31], and Bang *et al*. found that the patients with extensive flame burns are prone to septicemia [32]. In our study,patients with KP-BSIs, with the infection source from skin and soft tissue, were accepted to the burn center of the hospital, and all suffered from flame injuries. In 10 out of these 12 patients, burns were found on over 30% of total body surface area, and they underwent surgeries, usually comprised of debridement and grafting, and most of them received antibiotic therapies containing third or fourth generation cephalosporins or carbapenems. A previous 9-year-long study confirmed that CRKP incidences increase significantly in severe burn patients with late BSIs [33]. Additionally, Cen *et al*. determined that the rate of CRKP strains has increased in recent years, although *Pseudomonas aeruginosa* and *Acinetobacter baumannii* were more frequently detected gram-negative bacteria in a Chinese burn ward [34]. This suggests that it is vital to protect the integrity of skin and mucosal barriers in the critically ill patients or the patients undergoing invasive procedures, in order to prevent the development of antibiotic-resistant bacterial infections. All necessary contact precautions should be employed by hospital staff caring for these high-risk patients.

Interestingly, CSKP-BSIs were especially common among the patients with diabetes mellitus, which may be related to hypermucoviscous *K. pneumoniae* (HVKP). Diabetes mellitus has been considered a significant risk factor for HVKP, prevalent in the Asian Pacific Rim since the 1980s [35]. Our unpublished data showed a high rate of HVKP isolates in our hospital, and the overwhelming majority of them were CSKP.

The crude 28-day mortality in KP-BSI patients was shown to be 22.8%, which increased to 33.3% in CRKP-BSI patients, similar to the rates obtained in both Chinese and foreign studies [12, 25, 29, 36–38]. Furthermore, the mortality of CRKP-BSI patients was shown to be higher than that of CSKP-BSI patients, which may be a consequence of the infection with ST11 clone, which is a dominant CRKP strain in China [28] with an increased pathogenic potential and resistance to serum killing [39, 40]. Additionally, due to the limited CRKP treatment options, inappropriate empirical therapy may contribute to their deleterious outcomes [4, 19]. According to the recent clinical observations, the treatment of CRKP infections by a combination therapy may result in the reduced mortality compared with monotherapy [11, 12], but the treatment should be further optimized.

To further investigate the risk factors associated with crude 28-day mortality, we evaluated the influence of patient characteristics, *K. pneumoniae* isolates, and the therapeutic interventions on KP-BSIs. After adjusting for multiple confounders, several parameters, such as probable source of infection from lung and high SOFA score, were associated with a higher crude 28-day mortality, as previously described [9, 41, 42]. For patients with SOFA scores above 7 at the onset of bacteremia, the 14-day mortality rate was shown to increase [43]. Here, a median SOFA score at the onset of CRKP-BSI was 9. Therefore, SOFA score determined at the onset of BSI may capture the risk associated with the severity of BSI or the clinical impact of any other condition. It may help physicians identify the patients with BSIs who have a higher risk of in-hospital death. No significant difference was observed between the inappropriate empirical therapy and mortality, however, carbapenem resistance was not found to be an independent predictor of mortality, as previously reported [44], but such trend was observed, which may be explained by a relatively small sample population included in this study.

Increasing antibiotic resistance in ICUs represents a major clinical concern, and we showed here that almost half of all CRKP isolates were obtained from ICU patients, while further analysis showed that CRKP-BSIs are associated with ICU acquired infection, which may lead to higher mortality rates (38.5%). Different risk factors for BSI development may affect patients in ICUs, including the higher disease severity, disruption of anatomical barriers, and impaired immunological response [45], which may lead to the increase in the length of hospital stay, worse outcomes, and the difficulties in the treatment [46, 47]. Furthermore, we analyzed the genetic relatedness of CRKP strains isolated from ICU patients, showing that the randomly isolated CRKP strains are almost identical, which may result from a nosocomial clonal expansion. It is worth noting that the highest rate of CRKP-BSIs in ICU rose up to 80% in 2013, but later, this percentage apparently decreased, most likely because of an integrated stewardship prevention study that was conducted in our ICU from February 1, 2014, to August 31, 2015. In this period, infection-control measures, such as active surveillance culture, isolation precautions, enhanced contact precautions, disinfection, and sterilization were applied. Therefore, prompt and appropriate infection control should be implemented upon the admission of high-risk patients, especially in ICU.

Our study has several limitations. The extended spectrum ß lactamase-producing *K. pneumoniae* strains represent a major issue when analyzing multidrug-resistant *K. pneumoniae*, however, we have not discussed this in our study. Clinical data were obtained retrospectively from medical records, and therefore, some differences in physician practices or accuracy of information may exist. Additionally, the data for patients who may have had significant BSI symptoms such as septic shock and hyperpyrexia, but were not tested due to the patient refusal or because their blood culture was negative, were not included. Finally, this was a single-center study, including 114 patients with detailed clinical analysis, and further multi-centric, prospective studies are needed to confirm our findings and to assist physicians in adopting more effective approaches for KP-BSI treatment.

## Conclusions

A significant increase in the occurrence of CRKP among the identified KP-BSIs in our hospital was observed, especially in ICU since 2011. We demonstrated that skin and soft tissue infection source and ICU-acquired infection represent strong risk factors for CRKP-BSI development. Probable lung infection source and high SOFA score were independent predictors for crude 28-day mortality of KP-BSI patients. Taken together, our results clearly demonstrated that CRKP-BSIs are associated with high morbidity and mortality, and clone spread may contribute to the nosocomial dissemination of CRKP, requiring the implementation of appropriate infection control, especially in ICU.

## Abbreviations

BSI: Bloodstream infection
CCI: Charlson comorbidity index
CI: Confidence interval
CRKP: Carbapenem-resistant *Klebsiella pneumoniae*
CSKP: Carbapenem-sensitive *Klebsiella pneumoniae*
HVKP: Hypermucoviscous *Klebsiella pneumoniae*
ICU: Intensive care unit
IQR: Interquartile range
KP: *Klebsiella pneumoniae*
LOS: Length of stay
NA: Non-applicable
OR: Odds ratio
PBS: Pitt bacteraemia score
PFGE: Pulsed-field gel electrophoresis
SD: Standard deviation
SOFA score: Sequential organ failure assessment score
